# Trends of the Prevalence of Pre-gestational Diabetes in 2030 and 2050 in Belgrade Cohort

**DOI:** 10.3390/ijerph19116517

**Published:** 2022-05-27

**Authors:** Stefan Dugalic, Milos Petronijevic, Brankica Vasiljevic, Jovana Todorovic, Dejana Stanisavljevic, Aleksandra Jotic, Ljiljana Lukic, Tanja Milicic, Nebojsa Lalić, Katarina Lalic, Milica Stoiljkovic, Zorica Terzic-Supic, Tamara Stanisavljevic, Aleksandar Stefanovic, Katarina Stefanovic, Svetlana Vrzic-Petronijevic, Maja Macura, Igor Pantic, Pavle Piperac, Marija Jovanovic, Radmila Cerovic, Sinisa Djurasevic, Sandra Babic, Sonja Perkovic-Kepeci, Miroslava Gojnic

**Affiliations:** 1 Faculty of Medicine, Clinic for Obstetrics and Gynecology, University Clinical Centre of Serbia, University of Belgrade, 11000 Belgrade, Serbia; stef.dugalic@gmail.com (S.D.); ordinacija.petronijevic@gmail.com (M.P.); aleksandar.stefanovic@med.bg.ac.rs (A.S.); jeremick@hotmail.com (K.S.); vrzic.dr@gmail.com (S.V.-P.); maja_macura@live.com (M.M.); radojka.cerovic@gmail.com (R.C.); sandradrmilic@yahoo.com (S.B.); 2 Maternity and Child Health Service, NMC Royal Hospital DIP, Dubai Hospital, Dubai P.O. Box 7832, United Arab Emirates; brankica.vasiljevic1@gmail.com; 3 Faculty of Medicine, Institute of Social Medicine, University of Belgrade, 11000 Belgrade, Serbia; jovana.todorovic@med.bg.ac.rs (J.T.); zorica.terzic-supic@med.bg.ac.rs (Z.T.-S.); 4 Faculty of Medicine, Institute for Medical Statistics and Informatics, University of Belgrade, 11000 Belgrade, Serbia; dejana.stanisavljevic@med.bg.ac.rs; 5 Faculty of Medicine, Clinic for Endocrinology, Diabetes and Metabolic Diseases, University Clinical Centre of Serbia, University of Belgrade, 11000 Belgrade, Serbia; aleksandra.z.jotic@gmail.com (A.J.); ljlukic@eunet.rs (L.L.); icataca@gmail.com (T.M.); nebojsa.lalic@med.bg.ac.rs (N.L.); katarina.lalic@med.bg.ac.rs (K.L.); mmstoiljkovic@yahoo.com (M.S.); 6 Faculty of Medicine, University of Belgrade, 11000 Belgrade, Serbia; stamara8@yahoo.com; 7 Faculty of Medicine, Institute for Medical Physiology, University of Belgrade, 11000 Belgrade, Serbia; igor.pantic@med.bg.ac.rs; 8 Department for Humanities, University of Belgrade, Faculty of Medicine, 11000 Belgrade, Serbia; pavle.piperac@med.bg.ac.rs; 9 General Hospital Bor, 19210 Bor, Serbia; marija.jovanovic50.10@gmail.com; 10 Faculty of Biology, University of Belgrade, 11000 Belgrade, Serbia; sine@bio.bg.ac.rs; 11 General Hospital Pancevo, 26000 Pancevo, Serbia; perkovicsonja@yahoo.com

**Keywords:** forecasting, prevalence, pregnancy, diabetes, pre-gestational diabetes

## Abstract

The aim of this study was to analyze the trends in diabetes in pregnancy in Belgrade, Serbia for the period of the past decade and forecast the number of women with pre-gestational diabetes for the years 2030 and 2050. The study included the data on all pregnant women with diabetes from the registry of the deliveries in Belgrade, by the City Institute of Public Health of Belgrade, Serbia for the period between 2010 and 2020 and the published data on the deliveries on the territory of Belgrade. During the examined period the total number of live births in Belgrade was 196,987, and the prevalence of diabetes in pregnancy was 3.4%, with the total prevalence of pre-gestational diabetes of 0.7% and overall prevalence of GDM of 2.7%. The average age of women in our study was significantly lower in 2010 compared to 2020. The forecasted prevalence of pre-gestational diabetes among all pregnant women for 2030 is 2% and 4% for 2050 in our cohort. Our study showed that the prevalence of pre-gestational diabetes has increased both among all pregnant women and among women with diabetes in pregnancy in the past decade in Belgrade, Serbia and that it is expected to increase further in the next decades and to further double by 2050.

## 1. Introduction

More than 20 million births per year worldwide are associated with diabetes in pregnancy. The diabetes in pregnancy can be classified in two large categories: pre-gestational diabetes that includes mainly type 1 diabetes mellitus (T1DM), and type 2 diabetes mellitus (T2DM), along with specific types of diabetes and on the gestational diabetes mellitus (GDM), diabetes first diagnosed during the late second or third trimesters of pregnancy [1].

Although it is well known that both gestational diabetes (GDM) and pre-gestational diabetes are associated with numerous pregnancy complications, large studies that examine the prevalence of pre-gestational diabetes in the population are generally lacking. The majority of studies examining the characteristics of pregnant women with pre-gestational diabetes are based in clinical settings, and population-wide studies are sparse [2].

The vast majority of women with diabetes in pregnancy have GDM: between 80% and 90%, while the remaining 15% are the pre-gestational diabetes [1]. In past decades, the prevalence of pre-gestational diabetes is increasing mainly due to the rise of the prevalence of T2DM in the general population of women of the reproductive age, as a consequence of the obesity epidemic [3]. Pre-gestational diabetes is associated with the higher likelihood for congenital anomalies, stillbirth and large for gestational age infants, preterm births, and operative deliveries [3,4,5]. It is expected that the prevalence of pre-gestational diabetes during pregnancy reflects the prevalence of diabetes in the general population, but the women with pre-gestational diabetes have decreased fertility and the prevalence of pre-gestational diabetes underestimates the prevalence of the DM in the general population [1]. The decrease in fertility among women with pre-gestational diabetes is associated with the microvascular and cardiovascular complications among women with T1DM and with the high prevalence of obesity and polycystic ovarian syndrome among women with T2DM [1].

It is expected that the prevalence of overweight and obesity among the pregnant women in the future will increase, along with the increase in the body mass index of children and adolescents. The prevalence of the pre-gestational DM will also increase. All this raises more concerns from a public health perspective and the perspective of the possible pregnancy associated complications and their costs [1].

The total number of individuals with diabetes is now expected to increase to almost 600 million in the world by 2030 and 700 million by 2050, which is the double of the expected number for 2030 forecasted back in 2004 [6,7]. The rise in the prevalence of T2DM among younger populations has also shown that these women have similar rates of diabetic nephropathy, commonly considered the significant predictor of the adverse pregnancy outcomes, in women with T1DM in pregnancy. This is despite the fact that the women with T1DM have significantly longer duration of illness prior to pregnancy [8].

The health care system must be able to provide the identification of women with pre-gestational diabetes and to provide the adequate counseling to achieve the best possible time for conception at the time of the best possible glycemic control, enable the adequate control during pregnancy and puerperium in order to maximize the benefits for the mothers and for the newborns [1]. This can be achieved through the adequate allocation of the recourses in the health care system. Both material and human recourses need to be directed towards the women with diabetes [1,9,10].

In order for the health care system to provide those services, adequate planning is necessary based on the predicted number of women in need. The aim of this study is to analyze the trends in the diabetes in pregnancy in Belgrade, Serbia for the period of the past decade and to forecast the number of women with pre-gestational diabetes for the years 2030 and 2050.

## 2. Materials and Methods

The study included the data on all pregnant women with diabetes from the registry of the deliveries in Belgrade, by the City Institute of Public Health of Belgrade, Serbia for the period between 2010 and 2020 and the published data on the deliveries on the territory of Belgrade [11].

The data in the registry are the data on the maternal age, the health care institution that delivery occurred in, the newborns’ birth weight, the newborns’ birth length, the gestational age at birth, the Apgar score, the type of delivery, and the presence of any chronic illness of pregnant women (chronic hypertension, preeclampsia, pregnancy induced hypertension, development of the HEELP syndrome, diabetes type).

For the purpose of this study, we analyzed the trends in the number of women with pre-gestational diabetes and used the forecast analysis to predict the number of women with pre-gestational diabetes in 2030 and in 2050. Based on the reported number of deliveries in Belgrade in each year, we calculated the prevalence of pre-gestational diabetes and GDM among all pregnant women who delivered in Belgrade in each year.

The Ethical committee of the Faculty of Medicine, University of Belgrade approved the study (No 1322/IX-80).

Statistical analyses were done using the descriptive and analytical statistics. The differences between the groups on the numerical variables were examined using the univariate variance analysis (ANOVA). Time trends were analyzed creating the traditional forecasting models with the specification of the data of the final forecast of 2050. All statistical analyses were done using the SPSS for Windows 22.0.

## 3. Results

**Figure 1:**
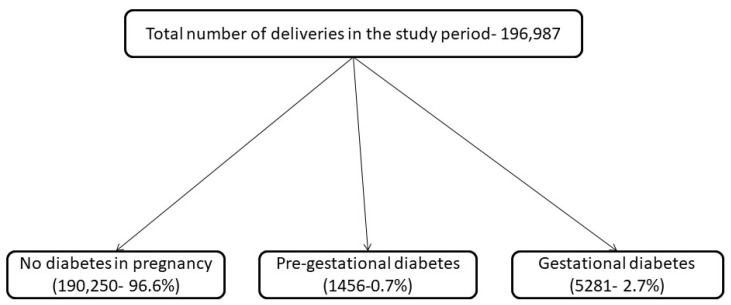
Flow diagram representing the study population.

During the examined period, the total number of live births in Belgrade was 196,987, and the prevalence of diabetes in pregnancy was 3.4%, with the total prevalence of pre-gestational diabetes of 0.7% and overall prevalence of GDM of 2.7%. The prevalence of diabetes in pregnancy and factors associated with it are described elsewhere [12]. Almost four fifths of the women with diabetes had GDM (5281—78.4%), just under one fifth (1318—19.6%) had T1DM, while 138 women (2.0%) had T2DM. The total number of women with pre-gestational diabetes was 1456 (21.6%). The highest number of women treated with pre-gestational diabetes was in 2018 (261 women) and the highest prevalence of pre-gestational diabetes among women with diabetes was 71.5% in 2020. A flow diagram showing the study population is presented in Figure 1.

The total number of women with pre-gestational and GDM and the prevalence of pre-gestational and GDM in each year of the study are presented in Table 1.

**Figure 2:**
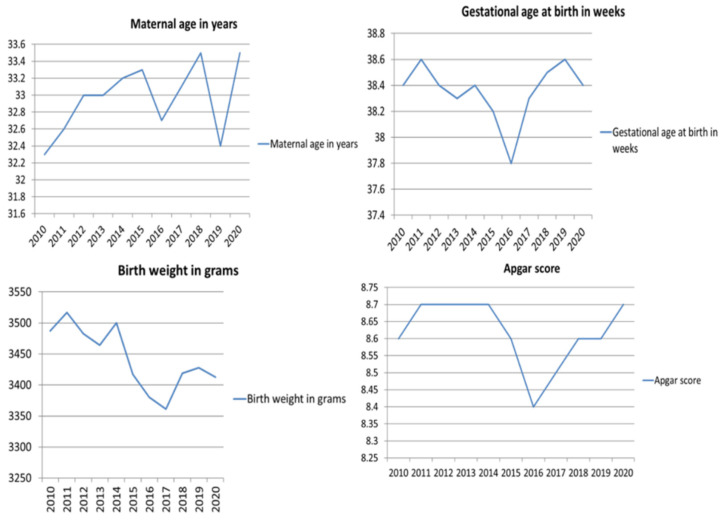
The trends in the maternal age, gestational age at delivery, newborns’ birth weight and Apgar scores in the study period.

The average age of women with DM included in the study was 32.96 ± 5.22 years, the average gestational age at delivery was 38.4 ± 1.86 weeks, the average newborns’ birth weight was 3453.99 ± 611.11 g and the average Apgar score was 8.63 ± 1.21. Maternal age increased in this period from 32.3 ± 5.23 to 33.5 ± 5.30 years, gestational age at delivery was the same in 2010 and in 2020 (38.4 weeks) Apgar score remained the same (8.60 ± 1.2 in 2010 and 8.68 ± 1.09), except for the decrease in 2016 (8.44 ± 1.43). In 2016 there was the lowest average gestational age at delivery (37.80 ± 2.22 weeks), birth weight (3380.27 ± 627.84) and the lowest average Apgar score (8.44 ± 1.43). The average maternal age differed significantly between 2010 and 2020 (*p* = 0.035). The trends in the maternal age, the gestational age at delivery, the newborns’ birth weight and the Apgar scores in the study period are presented in Figure 2.

**Figure 3:**
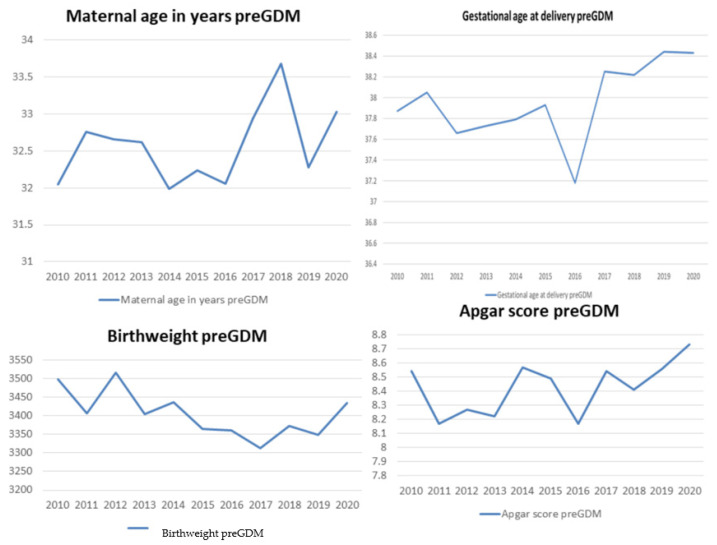
Trends in average maternal age, gestational age at delivery, newborns’ birth weight and average Apgar scores among women with pre-gestational diabetes.

There were no significant differences in the average maternal age, the average gestational age at birth, the average birth weight, or the average Apgar scores between the different years in our examined period among the women with pre-gestational diabetes. Trends in the average maternal age, the gestational age at delivery, the newborns’ birth weight and the average Apgar scores among women with pre-gestational diabetes are presented in Figure 3.

**Figure 4:**
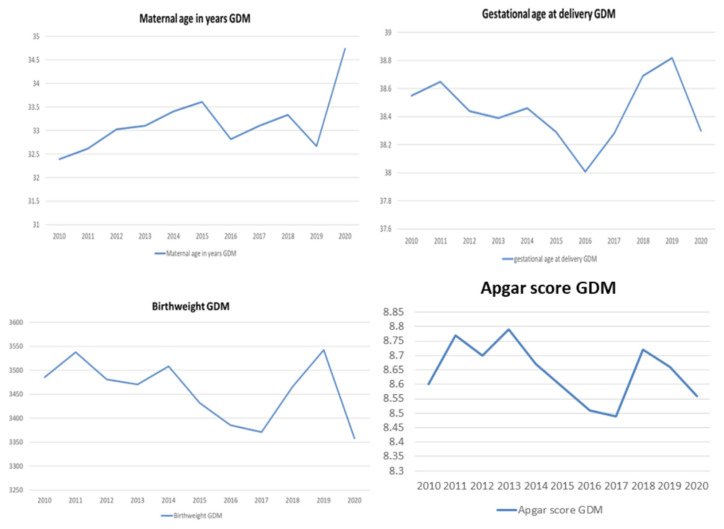
The trends in average maternal age, gestational age at delivery, newborns’ birth weight and average Apgar scores among women with GDM.

Women with GDM were significantly older in 2020 compared to women in 2010 and 2011 (33.74 ± 5.24 years in 2020, compared to 32.39 ± 5.18 in 2010, and 32.62 ± 5.09 in 2011, *p* = 0.003 and *p* = 0.013, respectively). Gestational age at delivery was the lowest in 2016, with significant differences between 2016 and 2010, 2011, 2012, 2013, 2014, 2018 and 2019. The trends in average maternal age, the gestational age at delivery, the newborns’ birth weight and the average Apgar scores among women with GDM are presented in Figure 4.

**Figure 5:**
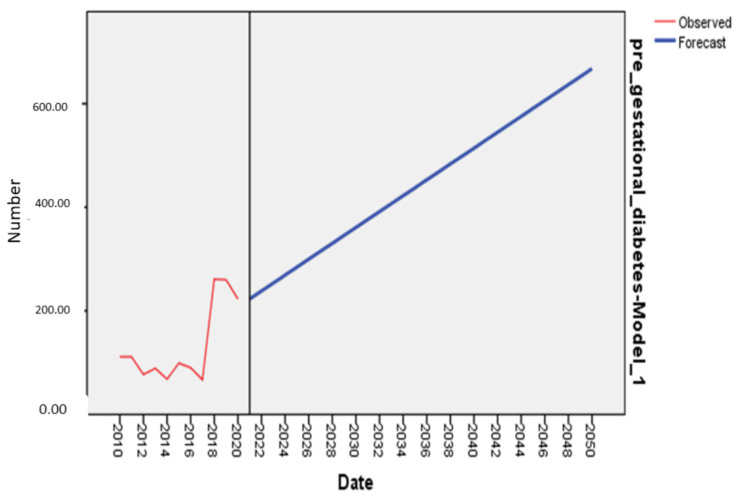
The forecast of the total number of women with pre-gestational diabetes in 2030 and 2050.

The forecasted number of women with GDM for 2030 is 180 (95% CI: −1290–1650). The forecasted number of women with pre-gestational diabetes for 2030 is 361 (95% CI: 225–497) and is 668 (95% CI: 532–803) for 2050 and the forecasted prevalence of pre-gestational diabetes among all pregnant women for 2030 is 2% and 4% for 2050. The forecast for pre-gestational diabetes is presented on Figure 5.

The model was assessed using the simulation performance parameters, the root mean square error (RMSE) was 60.04, mean absolute percentage error (MAPE) was 43.94, mean absolute error (MAE) was 46.86.

The forecasted number of live births for both 2030 and 2050 is 17908 (95% CI: 16,241–19,575) making the forecasted prevalence of pre-gestational diabetes 2% for 2030 and 3.7% for 2050.

## 4. Discussion

The aim of this study was to examine the trends and to forecast the number of women with pre-gestational diabetes using the registry data for the period of 11 years at the territory of the City of Belgrade, Serbia. Our study has shown that the prevalence of pre-gestational diabetes among the women with diabetes in Belgrade, Serbia increased in the past decade, as did the prevalence of pre-gestational diabetes among all pregnant women. Further, according to our results, the prevalence of pre-gestational diabetes among all pregnant women is expected to increase in the next decades to 2% in 2030 and almost 4% in 2050.

The increase in the prevalence of pre-gestational diabetes is in line with the increase in the prevalence of DM in all age groups in both sexes, including the increase among women of the reproductive age [6]. This increase is largely associated with the increase in the prevalence of obesity worldwide and as this increase is not expected to slow down in the next decades, the forecasted increase in the prevalence of pre-gestational diabetes is fitting to these expectations [13].

As pre-gestational diabetes is associated with the decreased fertility rates, the increase in the number of women in the reproductive age with diabetes may lead to increase in the needs for the assisted reproductive technologies, its costs and the burden of pregnancy risks associated with them [1]. Further, the pregnancies with diabetes significantly burden the healthcare system through the necessities for the strict control, for diagnosis and treatment of the pregnancy associated complications and for the various adverse pregnancy outcomes for both mothers and newborns [14].

Increase in the prevalence of pre-gestational diabetes is expected to lead to the increase in the prevalence of all associated pregnancy complications affecting the mothers and the newborns, such as miscarriages, Caesarean deliveries, lacerations during vaginal deliveries, venous thromboembolism, congenital malformations, perinatal asphyxia and an overall higher perinatal mortality [15,16,17]. All of these complications are associated with the higher likelihood for increase in the length of hospital stay and the increase in the health care required for each women and the newborn [18].

The health care systems must work toward being able to provide best possible pre-conception and pre-natal care for women with pre-gestational diabetes. In order to achieve that, there is a highlighted need for strengthening the surveillance system for diabetes in pregnancy, mainly through the improvement of existing data sources, improvement of data quality and diabetes screening. With this the researchers, public health professionals, clinicians and all stakeholders in the health care system can have better insight in the actual burden of diabetes in pregnancy and all of its complications.

The average age of women in our study was significantly lower in 2010 compared to 2020, which is in line with the increasing maternal age worldwide [19,20]. However, these differences were not present when the group of pregnant women with pre-gestational diabetes only was analyzed, and were present among women with GDM. The increasing maternal age is the risk factor for the development of GDM, while the women with pre-gestational diabetes may be more prone not to postpone the pregnancy, and to get pregnant at the point with the shorter duration of illness.

### Limitations

Our predictions are based on the citywide registry data and may not reflect the country level population. However, the majority of women with diabetes in pregnancy are referred to Belgrade for delivery from other regions in Serbia. Moreover, as shown previously, the analyses of the trends in the prevalence of diabetes in the general population and the forecasts based on these trends tend to underestimate the actual prevalence of the disease and the increase in diabetes prevalence is more than a double compared to the forecasted prevalence. This can significantly influence the clinicians working with the pregnant women with the pre-gestational diabetes and burden the healthcare system even further. We did not include the changes in the prevalence of the possible risk factors for diabetes in pregnancy in the forecasting analysis, and therefore did not account for the possible changes in those factors as well.

## 5. Conclusions

Our study showed that the prevalence of pre-gestational diabetes has increased both among all pregnant women and among women with diabetes in pregnancy in the past decade in Belgrade, Serbia and that it is expected to increase further in the next decades to 2% in 2030 and to further double by 2050 to 4%. As all types of hyperglycemia in pregnancy have significant intergenerational effect, expecting to influence the global prevalence of diabetes, as well, the forecasted increase in the prevalence of pre-gestational diabetes places high responsibility on the clinicians working with the women with diabetes in pregnancy as the adequate glycemic control not only influences the proximal outcomes related directly to the pregnancy and the childbirth, but also distal outcomes relating mostly to the risk for the diabetic complications in the women with pre-gestational diabetes.

## Figures and Tables

**Table 1:** The total number of women with pre-gestational diabetes and GDM and the prevalence of pre-gestational diabetes and GDM in each year of the study.

Year	Number of Women with Pregestational Diabetes	Prevalence of Pregestational Diabetes among DM in Pregnancy (%)	Prevalence of Pregestational Diabetes among All Pregnancies (%)	Number of Women with GDM	Prevalence of GDM among DM in Pregnancy (%)	Prevalence of GDM among All Pregnancies (%)
2010	111	15.30	0.6	614	84.7	3.4
2011	111	14.90	0.6	633	85.1	3.6
2012	77	8.50	0.4	831	91.5	4.5
2013	89	8.70	0.4	933	91.3	5.2
2014	68	11.70	0.4	513	88.3	2.8
2015	99	22.70	0.5	338	77.3	1.8
2016	90	19.70	0.5	367	80.3	2.0
2017	67	16.80	0.4	333	83.2	1.8
2018	261	36.70	1.4	450	63.3	2.5
2019	260	59.10	1.4	180	40.9	1.0
2020	223	71.50	1.4	89	28.5	0.6

## Data Availability

Not applicable. The data used in this study is the data owned by the City Institute of Public Health of Belgrade, Serbia.
